# Conspecific cueing or cooperative feeding?—Foraging stable flies are visually attracted to conspecific flies

**DOI:** 10.1111/mve.70057

**Published:** 2026-03-13

**Authors:** Emmanuel Hung, Justin Wong, Augustus Negraeff, Anya Gould, Gerhard Gries

**Affiliations:** ^1^ Department of Biological Sciences Simon Fraser University Burnaby British Columbia Canada

**Keywords:** Diptera, foraging, host selection, Muscidae, *Stomoxys calcitrans*

## Abstract

Stable flies, *Stomoxys calcitrans*, blood‐feed daily on livestock hosts. The presence of conspecific flies on host animals may signify host suitability (‘conspecific cueing’) or may signal density‐dependent feeding benefits (‘Allee effects’). However, stable flies captured on sticky traps reportedly render traps less attractive to conspecifics. We investigated whether this deterrent effect is context‐dependent, and whether host‐foraging stable flies are attracted to, rather than deterred by, conspecifics. In two‐choice laboratory bioassays, we offered food‐deprived, CO_2_‐stimulated stable flies a choice between paired landing platforms that were baited or not (control) with conspecific flies (‘bait flies’). The presence of bait flies—irrespective of their abundance, sex or relative position on the platform—prompted attraction and landing of foraging flies. Even heterospecific flies and fly‐look‐alike decoys were attractive, provided they visually resembled stable flies. As shown in separate bioassays, chemical cues of bait flies on their own were not attractive to foraging flies. Our data suggest that foraging stable flies may aggregate on hosts. Aggregated blood‐feeding may be adaptive in that aggregated conspecifics on a host animal may signal the presence of a suitable food resource, and group‐feeding may help suppress immune responses of host animals.

## Graphical abstract

- In two‐choice laboratory bioassays, food‐deprived and CO_2_‐stimulated stable flies were offered paired landing platforms that were baited or not (control) with conspecific flies.
- The presence of conspecifics prompted the attraction and landing of foraging stable flies. Even heterospecific flies and 3D‐printed decoys were attractive, provided they visually resembled stable flies.
- Conspecific fly presence on a host animal may signify host suitability (‘conspecific cueing’) or may signal density‐dependent feeding benefits (‘Allee effects’).

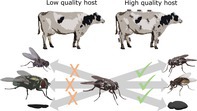

## INTRODUCTION

Individual animals making reproductive fitness‐related decisions may be attracted to, or repelled by, the presence of conspecifics (Prokopy & Roitberg, 2001). Attraction to conspecifics often results in aggregations of animals, a phenomenon that occurs across various animal taxa, including social and non‐social insects (Prokopy & Roitberg, 2001; Ward & Webster, 2016). Attraction to conspecifics is well documented in the context of habitat selection (Buxton et al., 2020) but also occurs in the context of mate‐location, oviposition, foraging and defence (Gascoigne et al., 2009; Gillmeister, 1999; Ruxton et al., 1995). Foraging animals accrue benefits by responding to the presence of conspecifics as indicators of resource patch quality (i.e., conspecific cueing) or by gaining population size‐dependent reproductive fitness benefits (i.e., Allee effects) (Allee, 1931; Donahue, 2006; Stephens et al., 1999).

Attraction to conspecifics is prevalent in dipterans. Visual and/or olfactory cues associated with conspecific adults, their eggs or their larvae affect oviposition site selection in muscoid flies (Baleba et al., 2020; Hoshizaki et al., 2020; Lam et al., 2007), tephritid fruit flies and drosophilid vinegar flies (Díaz‐Fleischer & Aluja, 2003; Mangel & Roitberg, 1989; Piñero & Prokopy, 2004; Prokopy et al., 2000). Dipterans form feeding aggregations as larvae (Durisko et al., 2014; Rivers et al., 2011) and as adults (Wertheim et al., 2005). The ‘fly factor phenomenon’ describes aggregative foraging behaviour of adult house flies, *Musca domestica*, green bottle flies, *Lucilia sericata*, and black blow flies, *Phormia regina*, in response to metabolites or microbial symbionts in the flies' faeces or regurgitate (Barnhart & Chadwick, 1953; Dethier, 1955; Holl & Gries, 2018; Uriel et al., 2020). Similarly, the ‘invitation effect’ refers to the phenomenon where host‐seeking mosquitoes and black flies preferentially seek host animals that are already being fed upon by conspecifics (Ahmadi & McClelland, 1985; Alekseev et al., 1977; McCall & Lemoh, 1997). Both the ‘fly factor phenomenon’ and the ‘invitation effect’ are thought to be mediated by cues or signals derived from conspecifics, or their metabolites, rather than the resource currently or previously fed on by dipterans.

The cues that invoke responses by foraging conspecific flies may have visual, chemical, acoustic and/or tactile characteristics (Parrish & Hamner, 1997; Ward & Webster, 2016). These cues, or signals, may originate from the flies themselves (e.g., Eichorn et al., 2017), or from their faeces, regurgitate or deposited eggs (Brodie et al., 2015; Holl & Gries, 2018; Lam et al., 2007). Foraging flies are attracted to aggregation pheromones produced by conspecific flies (Jaenike et al., 1992; Retkute et al., 2021; Schlein et al., 1984; Wertheim et al., 2005, 2006) and to semiochemicals (message‐bearing chemicals) emitted by the flies' bacterial symbionts (Brodie et al., 2015; Uriel et al., 2020; Venu et al., 2014; Zheng et al., 2013). The physical presence of conspecific flies on specific resources may also affect behavioural responses of foraging flies, but this has been shown only in the context of oviposition (e.g., Hoshizaki et al., 2020; Prokopy et al., 2000; Rull et al., 2003).

For blood‐feeding arthropods that rely on host animals as nutritional resources, conspecific cueing may contribute to both host location and selection of suitable on‐host feeding sites. Aggregative blood‐feeding behaviour occurs in various ticks (Norval et al., 1989; Rechav et al., 1976; Wang et al., 2001), and poultry red mites, *Dermanyssus gallinae* (Koenraadt & Dicke, 2010), bed bugs, *Cimex lectularius* (Crawley et al., 2024), black flies, *Simulium damnosum* (McCall & Lemoh, 1997), sand flies, *Lutzomyia longipalpis* (Schlein et al., 1984; Tripet et al., 2009), Highland midges, *Culicoides impunctatus* (Blackwell et al., 1994), and *Aedes* mosquitoes (Ahmadi & McClelland, 1985; Alekseev et al., 1977; Cavanagh & Townson, 1986; Charlwood et al., 2014). Haematophagous arthropods may aggregate on a host for mate‐location or for cooperative feeding benefits. For example, cooperatively feeding sand flies locally concentrate salivary injections, thereby decreasing salivary expenditure for each fly in that group and amplifying the effects of immunosuppressive and anti‐hemostatic factors present in the saliva (Ribeiro, 1987; Ribeiro & Francischetti, 2003), ultimately improving blood meal accessibility. The latter benefit may also decrease time spent feeding, thus reducing the risk of injury from host‐defensive behaviour (Gillett & Wigglesworth, 1997; Lehane, 2005).

In the current study, we investigated the effect of stable fly, *Stomoxys calcitrans*, presence on foraging decisions of conspecific flies. Stable flies are metropolitan blood‐feeding pests of livestock (Rochon et al., 2021). Ingesting blood meals is vital for stable fly survival and reproduction (Anderson, 1978; Foil & Hogsette, 1994). Through blood meals, stable flies obtain fuel for metabolism and nutrients for the production of eggs (females) and seminal fluid (males) (Anderson, 1978; Moobola & Cupp, 1978). Currently known stimuli that affect landing site selection by stable flies include semiochemicals that emanate from conspecific faeces (Carlson et al., 2000) and contact sex pheromones on the integument of flies (Harris et al., 1976; Muhammed et al., 1975; Sonnet et al., 1979; Uebel et al., 1975).

Conversely, stable flies avoid landing on adhesive traps featuring captured con‐ or heterospecific flies (Beresford & Sutcliffe, 2017). However, traps in the Beresford‐and‐Sutcliff study were placed next to a calf barn, where flies may have recently blood‐fed which likely would have affected their subsequent responses to conspecific cues. The propensity of flies to join, ignore or avoid conspecifics is often dependent upon their physiological state (Prokopy & Roitberg, 2001).

Here, we tested the hypothesis that the physical presence of stable flies on a site context‐dependently affects landing decisions by foraging conspecifics. We tested this hypothesis by modulating: (*i*) the physiological condition of foraging male and female flies (fed or not; CO_2_‐stimulated or not) and the status of flies used as baits (‘bait flies’: live or dead); (*ii*) visual characteristics of sites; (*iii*) bait fly abundance on sites; (*iv*) the sex and species of bait flies (stable flies, house flies, black blow flies and horn flies, *Haematobia irritans*); and (*v*) visual and chemical cues of natural or decoy bait flies.

## MATERIALS AND METHODS

### Experimental insects

A laboratory colony of stable flies was (re‐)established annually using wild flies collected from Eagle Acres Dairy and Pumpkin Patch (Langley, BC, CA), and the Dairy Education and Research Center of the University of British Columbia (Agassiz, BC, CA). Stable flies were reared in metal mesh cages (45 × 45 × 45 cm; BioQuip®, Compton, CA, USA) in an ER‐75 walk‐in growth chamber (Bio Chambers Inc., Winnipeg, MB, CA) maintained at 25 ± 1°C, 60% relative humidity (RH), and a 14:10 h (light:dark) photoperiod. Flies were fed daily on citrated bovine blood obtained from a local abattoir. Bioassay flies were either starved 20–24 h (Exps. 1–4, 6–37) or fed 3–6 h prior to experimentation (Exp. 5). All bioassay flies were 3 to 7 days old (i.e., days post eclosion) and were briefly cold‐anaesthetized for sex determination and sorting at least 1 h prior to release into a bioassay cage.

House flies, horn flies and black blow flies were also kept in metal mesh cages (BioQuip®) and held in a naturally lit insectary room kept at 23–25°C and 30%–40% RH. House flies were purchased from the Beneficial Insectary (Redding, CA, USA), and horn flies were obtained from the Veterinary Entomology Research Laboratory at New Mexico State University (Las Cruces, NM, USA). Blow flies, reared on bovine liver (Supreme Meat Supplies Ltd., Burnaby, BC, CA), were collected from a 15‐year‐old laboratory colony in the Gries‐laboratory (Simon Fraser University, Burnaby, BC, CA), originally established with flies shipped from the Department of Integrative Biology at the University of Windsor (Windsor, ON, CA). Horn flies were provisioned twice daily with citrated bovine blood. House flies and blow flies were provided with sugar (Rogers Sugar, Vancouver, BC, CA) and water ad libitum. In experiments, we used 4‐ to 7‐day‐old house flies and blow flies, and 1‐ to 4‐day‐old horn flies.

### General bioassay design

**FIGURE 1:**
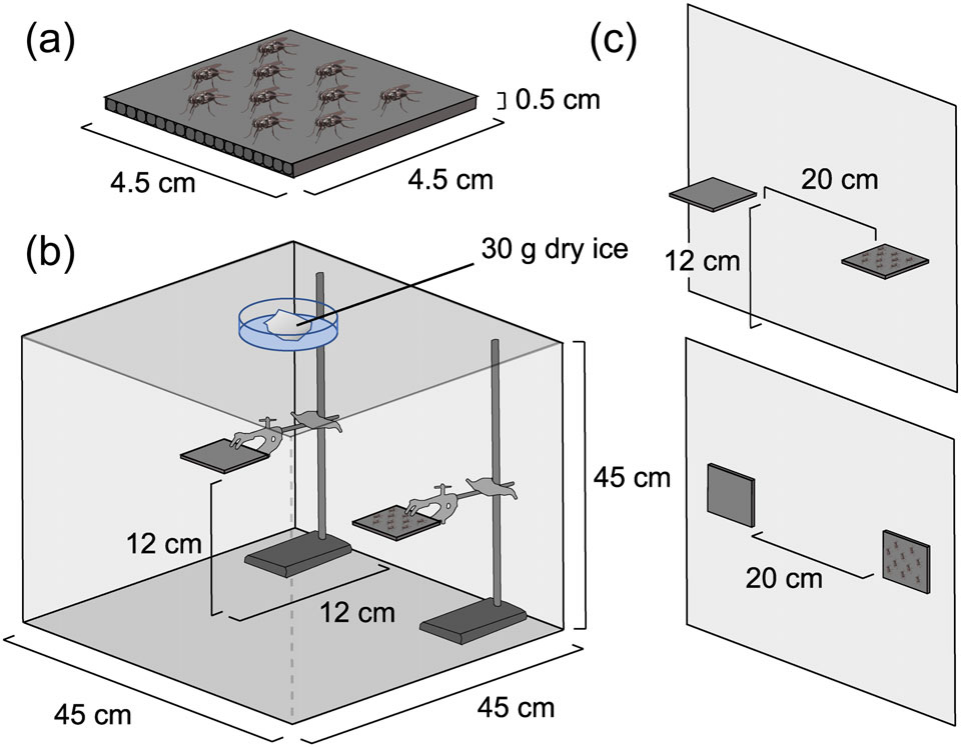
Diagrams illustrating the arrangement of design elements used for two‐choice experiments with stable flies. (a) Coroplast corrugated plastic square with a black poster board cover and glue‐mounted bait flies. (b) Bioassay cage with paired Coroplast squares as landing sites for flies (Exps. 1–9). (c) Paired squares mounted horizontally (top) or vertically (bottom) to an interior cage wall (Exps. 10–31, 33–35).

Two‐choice bioassays were run in the same type of cage used for fly rearing. To improve transmission of overhead light through the cage ceiling, the ceiling panel was replaced with a layer of fine white polyester diamond mesh (1 mm diam.; Fabricana, Coquitlam, BC, CA). The cage floor was lined with matte brown Kraft paper (NCR Corp., Duluth, GA, USA) to prevent unwanted light reflections. Two burette stands were placed in opposite corners of the cage's back wall (Figure 1b). A Coroplast square (4.5 cm × 4.5 cm × 0.5 cm) clamp‐mounted on each burette stand 12 cm above the cage floor served as a landing site for flies. The upper surface of each square was covered with black or white poster board (Staples, Framingham, MA, USA). In each replicate, the treatment square was baited with 10 flies (‘bait flies’) glue‐mounted on their ventral abdomen (LePage Ultra Gel super glue; Henkel Canada Corp., Mississauga, ON, CA) (Figure 1a). The paired control square received the equivalent amount of glue (used for mounting flies) which was deposited in places corresponding with those for mounting flies. Glue‐mounting bait flies did not affect their appearance or body posture. For each experimental replicate, 25 flies (‘bioassay flies’) were released into the cage and their landing responses on each Coroplast square were video‐recorded 5 min (C920x HD Pro Webcam; Logitech Canada Inc., Vancouver, BC, CA). To account for potential side bias, the position of the treatment square and the control square in the cage was alternated between replicates. All ‘bait flies’ and ‘bioassay flies’ were used only once and were cold‐euthanized after each replicate. The flies used in any experiment were collected from 2 to 6 fly colony cohorts and originated from several fly generations. For ‘parallel’ experiments, with replicates alternated between experiments, all flies in each set of parallel replicates originated from the same colony cohort. At least 10 replicates were run in each experiment (see Table 1).

**TABLE 1:** List of laboratory behavioural experiments^a^ (Exp.), the number of replicates (*n*) run and the paired stimuli presented to CO_2_‐stimulated^b^, food‐deprived^c^ bioassay stable flies^d^.

Exp. # (n)	Stimulus 1	Stimulus 2
Effects of sex (female or male) and condition (fed or not; CO_2_‐stimulated or not) of bioassay flies and status (live or dead) of bait flies on attraction of bioassay flies (Exps. 1–6)		
1 (12)	horizontal (horiz.) black square	horiz. black square with 10 live stable fly females
2 (12)	horiz. black square	horiz. black square with 10 live stable fly females
3 (10)	horiz. black square	horiz. black square with 10 live stable fly females
4 (11)	horiz. black square	horiz. black square with 10 live stable fly females
5 (10)	horiz. black square	horiz. black square with 10 live stable fly females
6 (10)	horiz. black square with 10 dead stable fly females	horiz. black square with 10 live stable fly females
Visual effects of site characteristics on attraction of bioassay flies (Exps. 7–12)		
7 (10)	horiz. black square	horiz. white square
8 (10)	horiz. black square with 10 dead stable fly females	horiz. white square with 10 dead stable fly females
9 (10)	horiz. white square	horiz. white square with 10 dead stable fly females
10 (10)	horiz. black square	vertical (vert.) black square
11 (10)	horiz. black square with 10 dead stable fly females	vert. black square with 10 dead stable fly females
12 (10)	vert. black square	vert. black square with 10 dead stable fly females
Effects of bait fly abundance on attraction of bioassay flies (Exps. 13–20)		
13 (10)	vert. black square	vert. black square with 1 dead stable fly female
14 (10)	vert. black square	vert. black square with 5 dead stable fly females
15 (10)	vert. black square	vert. black square with 10 dead stable fly females
16 (10)	vert. black square	vert. black square with 20 dead stable fly females
17 (10)	vert. black square with 10 dead stable fly females	vert. black square
18 (10)	vert. black square with 10 dead stable fly females	vert. black square with 1 dead stable fly female
19 (10)	vert. black square with 10 dead stable fly females	vert. black square with 5 dead stable fly females
20 (10)	vert. black square with 10 dead stable fly females	vert. black square with 20 dead stable fly females
Effects of bait fly sex and species on attraction of bioassay flies (Exps. 21–25)		
21 (10)	vert. black square	vert. black square with 10 dead stable fly females
22 (10)	vert. black square	vert. black square with 10 dead stable fly males
23 (10)	vert. black square	vert. black square with 10 dead house fly females
24 (10)	vert. black square	vert. black square with 10 dead horn fly females
25 (10)	vert. black square	vert. black square with 10 dead black blow fly females
Effects of bait fly visual cues on attraction of bioassay flies (Exps. 26–30)		
26 (10)	vert. black square with 10 dead stable fly females (head downward‐pointing)	vert. black square with 10 dead stable fly females (head upward‐pointing)
27 (10)	vert. black square with 10 dead stable fly females (mid‐legs ablated)	vert. black square with 10 dead stable fly females (wings ablated)
28 (10)	vert. black square	vert. black square with 10 dead stable fly females (mounted dorsal‐side‐up)
29 (10)	vert. black square	vert. black square with 10 dead stable fly females (mounted ventral‐side‐up)
30 (10)	vert. black square	vert. black square with 10 dead stable fly females (wings ablated)
Effects of bait fly chemical cues on attraction of bioassay flies (Exps. 31–32)		
31 (10)	vert. black square visually occluded by a perforated cover	vert. black square with 10 dead stable fly females visually occluded by a perforated cover
32 (10)	petri dish visually occluded by four layers of cheese cloth	petri dish with 10 live stable fly females visually occluded by four layers of cheese cloth
Effects of fly decoys in cage and room bioassays on attraction of bioassay flies (Exps. 33–37)		
33 (10)	vert. black square	vert. black square with 10 dead stable fly females
34 (10)	vert. black square	vert. black square with 10 3‐dimensional black plastic fly decoys
35 (10)	vert. black square	vert. black square with 10 2‐dimensional black plastic fly decoys
36 (10)	vert. black square	vert. black square with 31 dead stable fly females
37 (10)	vert. black square	vert. black square with 31 3‐dimensional black plastic fly decoys

^a^ Two‐choice bioassays were run in a metal mesh cage (45 × 45 × 45 cm; Figure 1b) (Exps. 1–31, 33–35), a 3‐chamber still‐air olfactometer (45 × 21 × 15 cm; Figure 9b) (Exp. 32), or a white‐walled room (2.9 m × 2.5 m × 2 m) (Exps. 36–37).

^b^ Flies were tested at elevated levels of CO_2_ released from dry ice (30 g) in an open petri dish in the centre of the cage ceiling (Figure 1b). Only in Exp. 4 were flies not CO_2_‐stimulated.

^c^ Bioassay flies were either starved 20–24 h (Exps. 1–4, 6–37) or fed 3–6 h prior to experimentation (Exp. 5).

^d^ All lab‐reared bioassay stable flies were 3‐ to 7‐day‐old (i.e., days post eclosion) at the time of testing. Groups of either female (Exps. 1, 3–37) or male (Exp. 2) bioassay flies were used.

### Specific experiments to test factors that may affect conspecific cueing of stable flies

#### Effects of sex (female or male) and condition (fed or not; CO_2_ ‐stimulated or not) of bioassay flies and status (live or dead) of bait flies (Exps. 1–6)

To test for attraction to conspecifics, 25 female bioassay flies (Exp. 1) and 25 male bioassay flies (Exp. 2) were offered a choice between paired squares (Figure 1), with 10 live female bait flies glue‐mounted on the treatment but not the control square (Table 1). In all subsequent experiments, only female bioassay flies were used. Because host‐foraging behaviours of biting flies are often CO_2_‐activated (Gatehouse and Lewis, 1973; Warnes and Finlayson, 1985), flies were tested in the presence of elevated CO_2_ levels originating from dry ice (30 g) in an open petri dish in the centre of the cage ceiling (Figure 1). Ambient CO_2_ levels (468 ± 7 ppm) and elevated CO_2_ levels (>5000 ppm) were measured using a Q‐Trak Indoor Air Quality Monitor (Model 7575; TSI Inc., Shoreview, MN, USA) whose sensor was positioned on the cage floor directly below the dry ice dish. Between bioassay replicates, the room air was vented out at least 5 min with a box fan.

To test whether attraction to conspecifics is contingent upon elevated levels of CO_2_ as a host indicator cue, parallel experiments 3 and 4 re‐tested the same paired stimuli as in experiment 1 but in the presence (Exp. 3) or absence (Exp. 4) of elevated CO_2_ levels (Table 1). In all prior and subsequent experiments, bioassay flies were CO_2_‐activated. To determine whether attraction to conspecifics is contingent upon the flies' feeding status, we also tested recently fed (3–6 h prior) bioassay flies that were not motivated to forage (Exp. 5). To determine whether flies were attracted only to live bait flies, bioassay flies were offered a choice between squares baited with 10 live or 10 dead glue‐mounted females (Exp. 6). The bait flies that had been cold‐euthanized (exposed to −15°C for 1 h) were allowed to thaw for 15 min at room temperature prior to bioassays. As bioassay flies did not distinguish between dead and live bait flies (see Results), all subsequent experiments used dead bait flies.

#### Visual effects of site characteristics (Exps. 7–12)

Because stable fly captures on white card traps deterred further fly captures (Beresford & Sutcliffe, 2017), and because foraging stable flies preferred dark objects to white objects as landing sites (Allan et al., 1987; Hung et al., 2024), we wanted to further determine whether attraction to conspecifics is affected by the reflective intensity of the landing site surface. To this end, foraging flies were offered a choice between a black and a white square, with neither square baited (Exp. 7), or both squares baited with 10 female flies each (Exp. 8, Table 1).

Flies were also offered a choice between two white squares, baited or not (control), with 10 female flies (Exp. 9).

As stable flies often blood‐feed while vertically oriented on the front legs of cattle (Bishopp, 1913; Rochon et al., 2021), we also offered foraging flies a choice between a horizontal and a vertical black square (Figure 1c), with neither square baited (Exp. 10), or both squares baited with 10 female flies each (Exp. 11, Table 1). As vertical squares were more attractive to foraging flies than horizontal squares (see Results), follow‐up experiment 12 tested the effect of two vertical black squares, with the treatment square, but not the control square, baited with 10 female flies. In this experiment and all subsequent experiments, paired vertically oriented black squares were affixed with copper wire to the interior cage wall, 12 cm above the cage floor (Figure 1c).

#### Effects of bait fly abundance (Exps. 13–20; S1‐S3)

To determine whether attraction to conspecifics requires a minimum abundance of conspecifics on sites, female bioassay flies were offered a choice between a vertical square without any flies and a vertical square baited with 1 (Exp. 13), 5 (Exp. 14), 10 (Exp. 15) or 20 (Exp. 16) female flies (Table 1). To further determine whether attraction to conspecifics increases with increasing abundance of conspecifics, bioassay flies were offered a choice between a vertical square baited with 10 female flies and a vertical square left unbaited (Exp. 17), or baited with 1 (Exp. 18), 5 (Exp. 19) or 20 (Exp. 20) female flies (Table 1).

To determine whether the orientation of squares (vertical or horizontal) alters the effect of bait fly abundance on attraction of conspecifics, bioassay flies were offered a choice between a horizontal (instead of a vertical) square baited with 10 female flies and a horizontal square baited with 5 female flies (Exp. S1) or 20 female flies (Exp. S2) (Table S1).

#### Effects of bait fly sex and species (Exps. 21–25)

To test whether attraction of stable flies to conspecifics is affected by the sex and species of bait flies, we offered female stable flies a choice between two vertically oriented black squares, with one square being unbaited and the other baited with 10 female or 10 male stable flies (Exps. 21, 22), 10 female house flies (Exp. 23), 10 female horn flies (Exp. 24) or 10 female black blow flies (Exp. 25) (Table 1).

#### Effects of bait fly visual cues (Exps. 26–30; S4‐S6)

To determine whether specific visual cues of conspecifics, such as body orientation and wing reflections, affect their attractiveness, we offered female bioassay flies a choice between two vertical black squares, each baited with 10 female flies that were (*i*) mounted dorsal‐side‐up with their heads facing either upward (characteristic of a feeding stable fly) (Rochon et al., 2021) or downward (Exp. 26) and (*ii*) mounted dorsal‐side‐up with their wings being either intact or ablated by micro‐scissors (Exp. 27) (Table 1). To account for the injurious effect of wing‐ablation, we removed the mid‐legs of wing‐intact flies.

To further investigate whether specific visual cues of conspecifics affect their attractiveness, follow‐up parallel experiments 28–30 (Table 1) offered bioassay flies a choice between two vertical black squares, one kept empty and the other baited with 10 flies that were mounted (*i*) dorsal‐side‐up and head upward‐facing (Exp. 28), (*ii*) ventral‐side‐up and head upward‐facing (Exp. 29) and (*iii*) dorsal‐side‐up, head upward‐facing and wings ablated (Exp. 30).

To determine whether the geometric orientation of bait flies modulated the attraction of conspecifics, female bioassay flies were offered a choice between a horizontal square with 10 female flies mounted dorsal‐side‐up and pointing randomly in various directions or with 10 female flies all pointing in the same (uniform) direction (Exp. S3, Table S1). To further determine potential interactive effects between the orientation of squares (vertical or horizontal), the status of bait flies (live or dead), their relative orientation on squares and the integrity of their wings on attraction of conspecifics, bioassay flies were offered a choice between two horizontal squares baited with live flies (Exp. S4) or dead flies (Exp. S5) that were glue‐mounted ventral‐side‐up or dorsal‐side‐up. Similarly, bioassay flies were offered a choice between two horizontal squares baited with live flies (Exp. S6) or dead flies (Exp. S7), that were glue‐mounted dorsal‐side‐up with wings intact or ablated (Table S1).

#### Effects of bait fly chemical cues (Exps. 31–32; S8)

To test whether chemical cues of conspecifics affect their attractiveness, we offered bioassay flies a choice between two squares—keeping the control square blank and baiting the treatment square with 10 female flies—and covered both the treatment and the control square with a perforated black poster board case to render the bait flies invisible (Exp. 31; Figure 9a) (Table 1).

Experiment 32 further tested whether bioassay flies are attracted to chemical rather than visual cues (or signals) of conspecific flies. The experimental design included a custom‐built two‐choice, still‐air, 3‐chamber plexiglass olfactometer (Figure 9b; Nayani et al., 2023) illuminated by an overhead metal halide lamp (MT400D/BUD/HTL‐BLUE, EYE Lighting Int., Mentor, OH, USA). The two lateral chambers of the olfactometer were each fitted with a glass petri dish which was baited, or not (control), with 10 live female flies. The flies' visual cues were occluded through four layers of white cheese cloth affixed to the perforated lid of both the baited and the unbaited petri dish. In each of 10 bioassay replicates, 15 food‐deprived bioassay flies were released into the central chamber of the olfactometer and allowed 6 h to respond to the conspecific flies in a lateral chamber. The bioassay flies could enter, but not exit, the lateral chambers through metal mesh funnels (Figure 7b). At the end of each replicate, the olfactometer was placed in a walk‐in freezer (−15°C) and the cold‐euthanized flies in each chamber were counted. The olfactometer was then washed with warm water and Sparkleen 1 detergent (Fisher Scientific, MA, USA), rinsed with distilled water and left to air dry overnight. The suitability of our bioassay design became evident in a pre‐screening experiment that offered a moist (treatment) or dry (control) cotton ball in lateral chambers and that demonstrated preferential entry of bioassay flies into the lateral chamber baited with the moist cotton ball (Table S1; Exp. S8; Figure S1).

#### Effects of fly decoys in cage and room bioassays (Exps. 33–37)

To rigorously compare the effects of stable flies against the effects of three‐dimensional (3D) or two‐dimensional (2D) wingless fly decoys of equivalent body size and shape, on the attraction of stable flies, parallel experiments 33–35 offered bioassay flies a choice between paired vertical black squares that were left unbaited or baited with 10 female stable flies (Exp. 33), 10 3D fly decoys (8 × 3.5 × 3 mm; Exp. 34) and 10 2D fly decoys (8 × 3.5 × 0.5 mm; Exp. 35) (Table 1). Decoys were designed with TinkerCAD (Autodesk Inc., Mill Valley, CA, USA) and printed using an Ender‐3 Pro 3D printer (Creality, Shenzhen, GD, CN) equipped with a black polyethylene terephthalate glycol (PETG) printing filament (1.75 mm; Amazon.com Inc., Seattle, WA, USA). The reflectance off the dorsal surface of a 3D decoy, 2D decoy and female stable fly (Figure S2) was measured with a JAZ spectrometer (Ocean Optics, Dunedin, FL, USA) calibrated with a 99% Spectralon reflectance standard (SRS‐99–010; Labsphere, North Sutton, NH, USA).

Drawing on results of experiments 33–35 obtained in bioassay cages, follow‐up parallel experiments 36 and 37 (Table 1) tested attraction of bioassay flies to bait stable flies (Exp. 36) and to 3D fly decoys (Exp. 37) in the much larger space of a room (2.9 m × 2 m × 2.5 m) with white walls. The room was fitted with two large Coroplast squares (25 cm × 25 cm × 0.5 cm) each covered with black poster board and taped 50 cm above the floor to opposite walls 2 m apart. An open petri dish containing CO_2_‐liberating dry ice (30 g) was mounted to the wall 10.5 cm above each square. For each replicate, a group of 50 bioassay flies was released from the room centre and offered a choice between an unbaited square and a square baited with 31 female stable flies (Exp. 36) or 31 3D fly decoys (Exp. 37). For each experimental replicate, the landing responses of flies on each square were video‐recorded for 5 min (C920x HD Pro Webcam; Logitech Canada Inc.). To account for potential side bias, the position of the treatment and the control square was alternated between replicates.

To further investigate whether the spatial arrangement of bait flies on squares in the bioassay room modulated the attraction of conspecifics, female bioassay flies were offered a choice between a square baited with 31 female flies mounted uniformly spaced over the entire square (‘uniform’) or mounted tightly clustered in the square centre (‘clustered’) (Exp. S9, Table S1). To determine whether wing‐derived visual cues of bait flies in a room setting affect their attractiveness, experiment S10 (Table S1) offered bioassay flies a choice between squares baited with 31 flies with wings intact or ablated.

### Statistical analyses

Data were analysed with R statistical software (4.1.2, R Core Team, 2021) using RStudio (v4.1.1 and older) and the packages *tidyr* and *dplyr*. Cage and room bioassay data were analysed with binomial generalized linear models using quasibinomial errors to account for overdispersion. For each experiment, we compared an intercept‐only model against a null model with a likelihood ratio test, allowing us to determine significant differences (*p* < 0.05) between landing responses on paired squares. Three‐chamber olfactometer data were analysed using a two‐sample *t*‐test. For all experiments, assumptions of data normality and homoscedasticity were tested, and model residuals were inspected.

## RESULTS

### Effects of bioassay fly sex (female or male) and condition (fed or not; CO_2_‐stimulated or not) and of bait fly status (live or dead) (Exps. 1–6)

**FIGURE 2:**
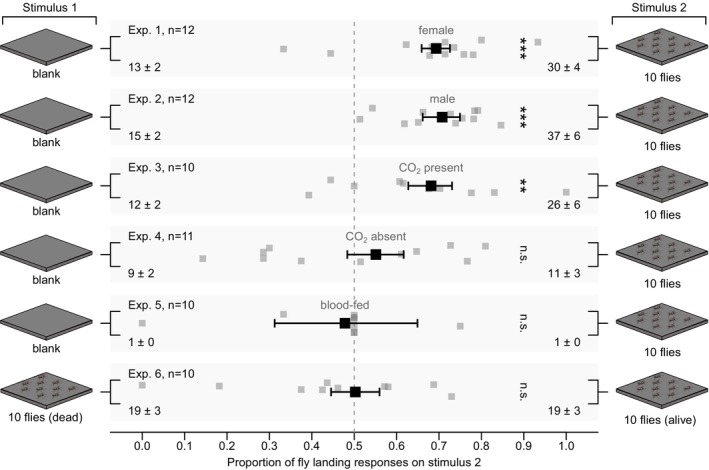
Effect of female stable fly presence on foraging decisions of stable flies. In each 5‐min experimental replicate (*n*), 25 flies were introduced into a bioassay cage and their landing responses on paired Coroplast squares (Figure 1a,b) were recorded. Foraging flies were food‐deprived females (Exps. 1, 3, 4, 6) or males (Exp. 2), and recently blood‐fed females (Exp. 5). Experiments 1–5 offered a choice between squares baited with 10 live female stable flies or kept unbaited. Experiment 6 offered a choice between squares baited with 10 live females or with 10 dead females that had been cold‐euthanized <1 h prior to bioassays. To stimulate foraging behaviour, dry ice (30 g) releasing CO_2_ as a host cue was placed above the bioassay cage in all experiments except experiment 4. Small grey symbols show the data of individual replicates and large black symbols the mean (±SEM). Mean absolute numbers (±SEM) of landing responses are listed at the bottom of each jitter plot. Asterisks (*) denote a significant preference for stimulus 2 (***p < 0.001, **p < 0.01; ‘n. s.’ = not significant, *p* > 0.05; likelihood ratio tests).

When CO_2_‐stimulated female (Exp. 1) and male (Exp. 2) bioassay flies were offered a choice between an unbaited square and a square baited with 10 female flies, both female and male bioassay flies landed more frequently on the squares with bait flies [Exp. 1 (*n* = 12): 30 ± 4 vs. 13 ± 2; likelihood ratio test, *F* = 30.892, *p* = 0.0002; Exp. 2 (*n* = 12): 37 ± 6 vs. 15 ± 2; *F* = 47.770, *p* = 0.0001; Figure 2], indicating attraction to conspecifics.

When CO_2_ was presented at an elevated level (Exp. 3; all other experiments), bioassay flies landed more often on the square with 10 female bait flies than on the empty control square [Exp. 3 (*n* = 10): 26 ± 6 vs. 12 ± 2; *F* = 13.903, *p* = 0.0047]. Conversely, when CO_2_ was presented at ambient level (Exp. 4), bioassay flies landed equally often on both squares regardless of whether or not they were baited with female flies [Exp. 4 (*n* = 11): 11 ± 3 vs. 9 ± 2; *F* = 0.615, *p* = 0.4512; Figure 2]. These results revealed that attraction to conspecifics occurred only under elevated levels of CO_2_, suggesting that host‐associated cues enhance the flies' responsiveness.

Recently blood‐fed (3–6 h prior) bioassay flies flew actively in the bioassay cage (EH pers. obs.) but rarely landed on squares, regardless of female bait flies mounted or not on squares [Exp. 5 (*n* = 10): 0.4 ± 0.3 vs. 0.5 ± 0.3; *F* = 0.068, *p* = 0.8183; Figure 2]. These results indicated that only food‐deprived flies are attracted to conspecific flies.

When bioassay flies were offered a choice between two squares that were baited with either live or dead female flies, bioassay flies landed equally often on each of the two squares [Exp. 6 (*n* = 10): 19 ± 3 vs. 19 ± 3; *F* = 0.027, *p* = 0.8750; Figure 2], indicating that both live and dead female flies are attractive to conspecifics.

### Effects of site characteristics (Exps. 7–12)

**FIGURE 3:**
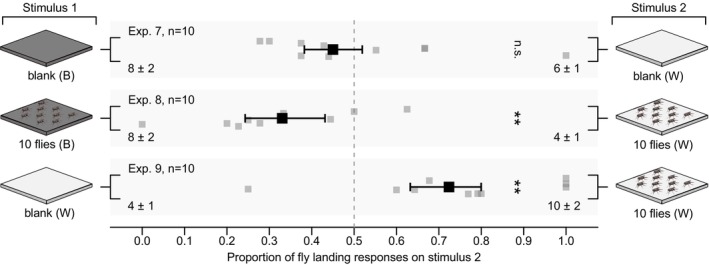
Effects of square reflective intensity and female stable fly presence on foraging decisions of female stable flies. In each 5‐min experimental replicate (*n*), 25 food‐deprived, CO_2_‐stimulated flies were introduced into a bioassay cage and their landing responses on paired Coroplast squares (Figure 1a,b) were recorded. Experiments 7 and 8 offered a choice between black (B) and white (W) squares that were baited, or not (blank control), with 10 glue‐mounted female flies. Experiment 9 offered white squares that were baited, or not (blank), with 10 glue‐mounted female flies. Small grey symbols show the data of individual replicates and large black symbols the mean (± SEM). Mean absolute numbers (± SEM) of landing responses are listed at the bottom of each jitter plot. Asterisks (*) denote a significant preference for a stimulus (**p < 0.01; ‘n. s.’ = not significant, *p* >0.05; likelihood ratio tests).

When bioassay flies were presented with a black and a white square, both unbaited (Exp. 7), or both baited with 10 female flies (Exp. 8), bioassay flies landed equally often on black and on white squares in the absence of bait flies [Exp. 7 (*n* = 10): 8 ± 2 vs. 6 ± 1; *F* = 1.3058, *p* = 0.2826; Figure 3] but landed more often on black squares than on white squares in the presence of bait flies [Exp. 8 (*n* = 10): 8 ± 2 vs. 4 ± 1; *F* = 11.081, *p* = 0.0088; Figure 3]. These data revealed an interaction between landing site characteristics and the presence of conspecifics on stable fly attraction.

When bioassay flies were presented with two white squares, one baited with 10 female flies and the other unbaited, bioassay flies landed more often on fly‐baited squares [Exp. 9 (*n* = 10): 10 ± 2 vs. 4 ± 1; *F* = 19.421, *p* = 0.0017; Figure 3], indicating that the presence of conspecific flies enhanced the attractiveness of white squares.

**FIGURE 4:**
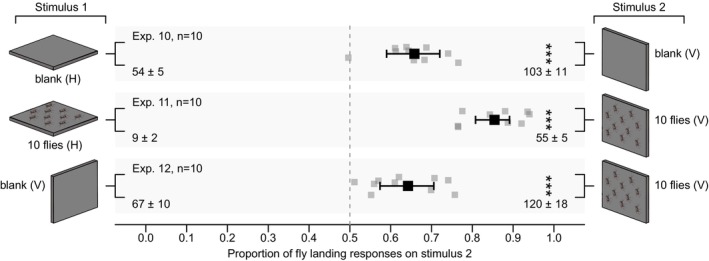
Effects of square orientation and female stable fly presence on foraging decisions of female stable flies. In each 5‐min experimental replicate (*n*), 25 food‐deprived, CO_2_‐stimulated flies were introduced into a bioassay cage and their landing responses on paired Coroplast squares (Figure 1a,c) were recorded. Experiments 10 and 11 offered a choice between horizontally (H) and vertically (V) oriented squares in the absence (Exp. 10), and presence (Exp. 11), of 10 glue‐mounted female flies on each square. Experiment 12 offered a choice between two vertical squares, one baited with 10 glue‐mounted female flies. Small grey symbols show the data of individual replicates and large black symbols the mean (±SEM). Mean absolute numbers (±SEM) of landing responses are listed at the bottom of each jitter plot. Asterisks (*) denote a significant preference for stimulus 2 (***p < 0.001; likelihood ratio tests).

When bioassay flies were offered a choice between two black squares that were oriented vertically or horizontally but not baited with flies (Exp. 10), or that both were baited with flies (Exp. 11), bioassay flies landed more often on vertically oriented squares [Exp. 10 (*n* = 10): 103 ± 11 vs. 54 ± 5; *F* = 41.488, *p* = 0.0001; Exp. 11 (*n* = 10): 55 ± 5 vs. 9 ± 2; *F* = 148.57, *p* < 0.0001; Figure 4], indicating a preference for vertical landing sites.

When female bioassay flies were offered a choice between two vertically oriented black squares, one baited with 10 female flies and the other unbaited, bioassay flies landed more often on fly‐baited squares [Exp. 12 (*n* = 10): 120 ± 18 vs. 67 ± 10; *F* = 25.157, *p* = 0.0007; Figure 4], indicating that conspecific presence enhanced the attractiveness of vertical landing sites.

### Effects of bait fly abundance (Exps. 13–20; S1–S3)

**FIGURE 5:**
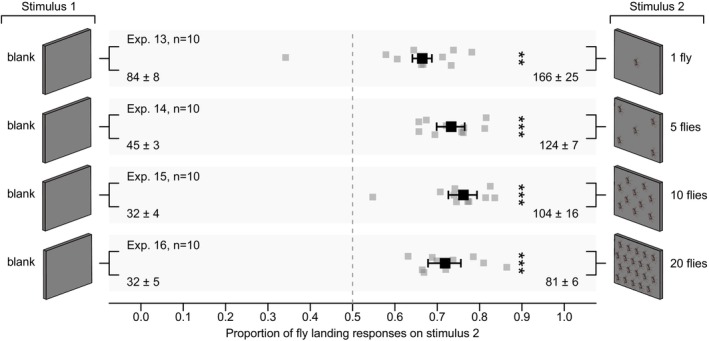
Effects of female stable fly abundance on squares on foraging decisions of female stable flies. In each 5‐min experimental replicate (*n*), 25 food‐deprived, CO_2_‐stimulated flies were introduced into a bioassay cage and their landing responses on paired Coroplast squares (Figure 1A,C) were recorded. Flies were offered a choice between squares that were left unbaited (blank) or baited with 1, 5, 10 or 20 glue‐mounted female flies. Small grey symbols show the data of individual replicates and large black symbols the mean (± SEM). Mean absolute numbers (±SEM) of landing responses are listed at the bottom of each jitter plot. Asterisks (*) denote a significant preference for stimulus 2 (***p <0.001, **p < 0.01; likelihood ratio tests).

When bioassay flies were offered a choice between an unbaited vertical square and a vertical square baited with 1 female fly (Exp. 13), 5 females (Exp. 14), 10 females (Exp. 15) or 20 females (Exp. 16), bioassay flies landed more often on fly‐baited squares, regardless of fly abundance [Exp. 13 (*n* = 10): 166 ± 25 vs. 84 ± 8; *F* = 19.802, *p* = 0.0016; Exp. 14 (*n* = 10): 124 ± 7 vs. 45 ± 3; *F* = 121.270, *p* < 0.0001; Exp. 15 (*n* = 10): 104 ± 16 vs. 32 ± 4; *F* = 99.617, *p* < 0.0001; Exp. 16 (*n* = 10): 81 ± 6 vs. 32 ± 5; *F* = 81.273, *p* < 0.0001; Figure 5].

**FIGURE 6:**
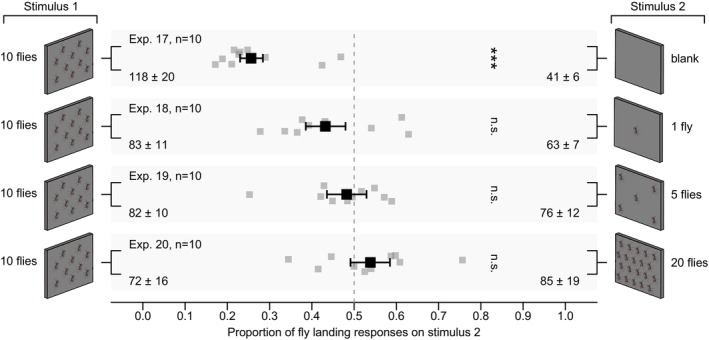
Effects of female stable fly abundance on squares on foraging decisions of female stable flies. In each 5‐min experimental replicate (*n*), 25 food‐deprived, CO_2_‐stimulated flies were introduced into a bioassay cage and their landing responses on paired Coroplast squares (Figure 1a,c) were recorded. Flies were offered a choice between squares that were baited with 10 *versus* 0 (blank), 1, 5 or 20 glue‐mounted female flies. Small grey symbols show the data of individual replicates and large black symbols the mean (± SEM). Mean absolute numbers (± SEM) of landing responses are listed at the bottom of each jitter plot. Asterisks (*) denote a significant preference for a stimulus (***p < 0.001; ‘n. s.’ = not significant, *p* > 0.05; likelihood ratio tests).

When female bioassay flies were offered a choice between a vertical square baited with 10 female flies and a vertical square that was unbaited (Exp. 17), or baited with 1 female (Exp. 18), 5 females (Exp. 19) or 20 females (Exp. 20), bioassay flies landed more often on the square baited with 10 flies than on the unbaited square [Exp. 17 (*n* = 10): 118 ± 20 vs. 41 ± 6; *F* = 50.431, *p* = 0.0001], but landed equally often on either square when both were fly‐baited regardless of fly abundance on squares [Exp. 18 (*n* = 10): 83 ± 11 vs. 63 ± 7; *F* = 3.721, *p* = 0.0858; Exp. 19 (*n* = 10): 82 ± 10 vs. 76 ± 12; *F* = 0.465, *p* = 0.5123; Exp. 20 (*n* = 10): 72 ± 16 vs. 85 ± 19; *F* = 1.895, *p* = 0.2019; Figure 6]. Similarly, when bioassay flies were offered a choice between two horizontal (instead of vertical) squares, one baited with 10 female flies and the other with either 5 females (Exp. S1) or 20 females (Exp. S2), bioassay flies landed equally often on either of the two squares in both experiments (Figure S3). All data combined indicate that it is the presence of flies, but not their abundance, that enhances the attraction of conspecifics.

### Effects of bait fly sex and bait fly species (Exps. 21–25)

**FIGURE 7:**
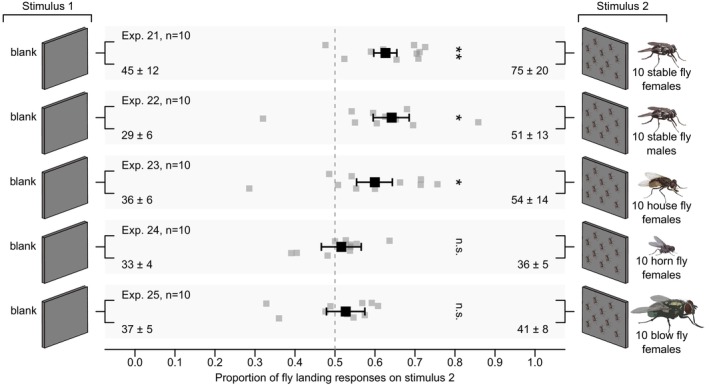
Effects of fly sex, and species, present on squares on foraging decisions of female stable flies. In each 5‐min experimental replicate (*n*), 25 food‐deprived, CO_2_‐stimulated flies were introduced into a bioassay cage and their landing responses on paired Coroplast squares (Figure 1a,c) were recorded. Experiments offered choices between squares that were baited, or not (blank), with 10 glue‐mounted stable fly females (Exp. 21), 10 stable fly males (Exp. 22), 10 house fly females (Exp. 23), 10 horn fly females (Exp. 24) and 10 black blow fly females (Exp. 25). Small grey symbols show the data of individual replicates and large black symbols the mean (± SEM). Mean absolute numbers (± SEM) of landing responses are listed at the bottom of each jitter plot. Asterisks (*) denote a significant preference for a stimulus (**p < 0.01, *p < 0.05; ‘n. s.’ = not significant, *p* > 0.05; likelihood ratio tests).

When female bioassay stable flies were offered a choice between two squares, one kept unbaited and the other baited with 10 female stable flies (Exp. 21) or 10 male stable flies (Exp. 22), bioassay flies landed more often on fly‐baited squares irrespective of bait fly sex [Exp. 21 (*n* = 10): 75 ± 20 vs. 45 ± 12; *F* = 21.589, *p* = 0.0012; Exp. 22 (*n* = 10): 51 ± 13 vs. 29 ± 6; *F* = 8.657, *p* = 0.0164; Figure 7], indicating that the sex of flies is irrelevant for the attraction of conspecifics.

When female bioassay stable flies were presented with two squares, one kept unbaited and the other baited with 10 female house flies (Exp. 23), 10 female horn flies (Exp. 24) or 10 female black blow flies (Exp. 25), bioassay stable flies landed more often on house fly‐baited squares than on unbaited control squares (Exp. 23 (*n* = 10): 54.5 ± 44.9 vs. 36.4 ± 18.3; *F* = 6.094, *p* = 0.0357; Figure 5), but landed equally often on horn fly‐ or on blow fly‐baited squares as on corresponding unbaited control squares [Exp. 24 (*n* = 10): 41.1 ± 26.6 vs. 36.9 ± 16.5; *F* = 0.876, *p* = 0.3737; Exp. 25 (*n* = 10): 35.6 ± 15.3 vs. 33.4 ± 11.8; *F* = 0.409, *p* = 0.5383; Figure 7]. In combination, these data suggest that female house flies, but not female horn flies or black blow flies, sufficiently resemble female stable flies for attraction of foraging stable flies.

### Visual effects of bait flies (Exps. 26–30)

**FIGURE 8:**
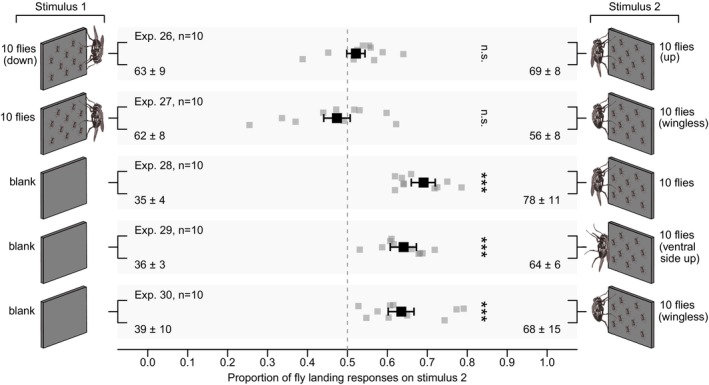
Visual effects of female stable flies mounted on squares on foraging decisions of female stable flies. In each 5‐min experimental replicate (*n*), 25 food‐deprived, CO_2_‐stimulated flies were introduced into a bioassay cage and their landing responses on paired Coroplast squares (Figure 1a,c) were recorded. Experiments 26 and 27 offered choices between two vertical black squares baited with 10 female flies, with their heads facing upward or downward (Exp. 26), and with their wings intact or ablated (Exp. 27). Parallel experiments 28–30 offered choices between two vertical squares, one not baited (blank) and the other baited with 10 female flies mounted dorsal‐side‐up (Exp. 28), ventral‐side‐up (Exp. 29) or dorsal‐side‐up with wings ablated (Exp. 30). Small grey symbols show the data of individual replicates and large black symbols the mean (±SEM). Mean absolute numbers (±SEM) of landing responses are listed at the bottom of each jitter plot. Asterisks (*) denote a significant preference for a stimulus (***p < 0.001; ‘n. s.’ = not significant, *p* > 0.05; likelihood ratio tests).

When foraging female bioassay flies were offered a choice between two vertical squares each baited with 10 female conspecifics but with their heads facing upward or downward (Exp. 26), or with their wings intact or ablated (Exp. 27), bioassay flies landed equally often on either of the two squares in each experiment [Exp. 26 (*n* = 10): 63 ± 9 vs. 69 ± 8; *F* = 1.035, *p* = 0.3355; Exp. 27 (*n* = 10): 62 ± 8 vs. 56 ± 8; *F* = 0.643, *p* = 0.4432; Figure 8], indicating that neither the orientation of bait flies nor the presence of their wings affected their attractiveness to foraging stable flies. Similarly, mounting bait flies with their wings intact or ablated, and with their body oriented in various ways, on horizontal instead of vertical squares, elicited indiscriminate landing responses by bioassay flies (Exps. S3–S7; Figure S4).

When female bioassay flies were offered a choice between two squares, one unbaited and the other baited with 10 female flies that were mounted dorsal‐side‐up/head upward‐facing (Exp. 28), ventral‐side‐up/head upward‐facing (Exp. 29) or dorsal‐side‐up/head upward‐facing/wings ablated (Exp. 30), bioassay flies in each of experiments 21–23 landed more often on fly‐baited squares than on unbaited control squares [Exp. 28 (*n* = 10): 78 ± 11 vs. 35 ± 4; *F* = 90.047, *p* < 0.0001; Exp. 29 (*n* = 10): 64 ± 6 vs. 36 ± 3; *F* = 57.102, *p* < 0.0001; Exp. 30 (*n* = 10): 68 ± 15 vs. 39 ± 10; *F* = 24.527, *p* = 0.0008; Figure 8]. All data combined indicate that a specific body position, body orientation and intact wings are not essential for attraction of conspecific flies.

### Chemical effects of bait flies (Exps. 31–32)

**FIGURE 9:**
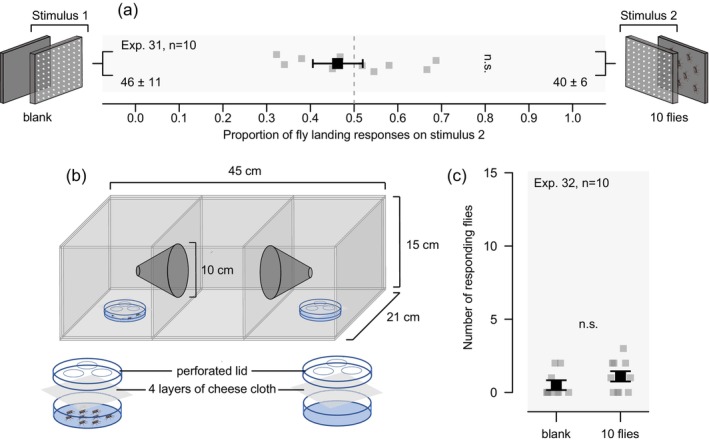
Olfactory effects of female stable flies on foraging decisions of female stable flies. (a) Landing responses, over 5‐min experimental replicates (*n*), of 25 food‐deprived, CO_2_‐stimulated flies introduced into a bioassay cage and presented with paired Coroplast squares (Figure 1a,c) that were baited, or not baited (blank), with 10 glue‐mounted female flies visually occluded with perforated black poster board. Mean absolute numbers (± SEM) of landing responses on squares are listed at the bottom of the jitter plot. (b) Graphical illustration of a 3‐chamber still‐air olfactometer used to test attraction of flies to fly odour emanating from cheesecloth‐covered petri dishes constraining flies. (C) Lack of stable fly attraction to stable fly odour (Exp. 32); in each 6‐h experimental replicate (n), 15 food‐deprived female flies were introduced into the central chamber of the olfactometer from which they could enter, but not exit, lateral chambers that were baited, or not (blank), with fly odour (flies residing visually occluded in a Petri dish). Small grey symbols show the data of individual replicates and large black symbols the mean (± SEM); ‘n. s.’ denotes no significant preference [*p* > 0.05; likelihood ratio test (Exp. 31); two‐sample *t*‐test (Exp. 32)].

When female bioassay flies were presented with a choice between two visually occluded squares, one unbaited and the other baited with 10 flies, bioassay flies landed equally often on either square [Exp. 31 (*n* = 10): 46 ± 11 vs. 40 ± 6; *F* = 0.908, *p* = 0.3656; Figure 9a]. Likewise, in still‐air olfactometer tests (Figure 9b), there was no significant difference in the number of bioassay flies entering the lateral chamber that was baited with 10 live (but visually occluded) female flies or that was left unbaited [Exp. 32 (*n* = 10): 1 ± 0 vs. 1 ± 0; *t*(9) = −1.152, *p* = 0.2789; Figure 9c]. Together, these data indicate that conspecific‐derived odours, on their own, do not attract stable flies.

### Effect of fly decoys in cage and room bioassays (Exps. 33–37)

**FIGURE 10:**
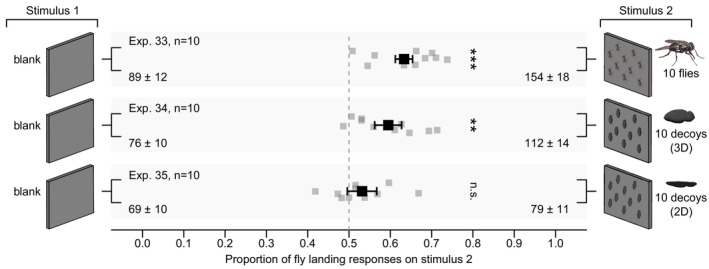
Effects of female stable flies and of fly decoys mounted on squares on foraging decisions of female stable flies. In each 5‐min experimental replicate (*n*), 25 food‐deprived, CO_2_‐stimulated female flies were introduced into a bioassay cage and their landing responses on paired Coroplast squares (Figure 1a,c) were recorded. Parallel experiments 33–35 offered flies choices between squares that were not baited (blank) or baited with 10 glue‐mounted female stable flies (Exp. 33), wingless 3‐dimensional (3D) fly decoys (Exp. 34) or wingless 2‐dimensional (2D) fly decoys (Exp. 35). Small grey symbols show the data of individual replicates and large black symbols the mean (± SEM). Mean absolute numbers (± SEM) of landing responses are listed at the bottom of each jitter plot. Asterisks (*) denote a signifiant preference for a stimulus (***p < 0.001, **p < 0.01; ‘n. s.’ = not significant, *p* > 0.05; likelihood ratio tests).

When female bioassay flies in parallel experiments 33–35 were offered a choice between two squares, one unbaited and the other baited with 10 flies (Exp. 33), 10 3D fly decoys (Exp. 34) or 10 2D fly decoys (Exp. 35), bioassay flies landed more often on squares baited with flies or with 3D fly decoys [Exp. 33 (*n* = 10): 154 ± 18 vs. 89 ± 12; *F* = 29.073, *p* = 0.0004; Exp. 34 (*n* = 10): 112 ± 14 vs. 76 ± 10; *F* = 14.647, *p* = 0.0040; Figure 10], but landed equally often on unbaited control squares and on squares baited with 2D fly decoys [Exp. 35 (*n* = 10): 79 ± 11 vs. 69 ± 10; *F* = 1.935, *p* = 0.1977; Figure 10].

**FIGURE 11:**
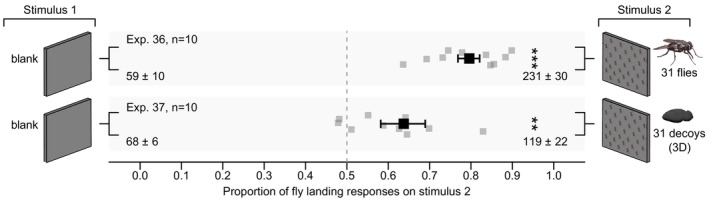
Effects of female stable flies and of fly decoys mounted on squares on foraging decisions of female stable flies in room (instead of cage) bioassays. In each 5‐min experimental replicate (*n*), 50 food‐deprived, CO_2_‐stimulated female flies were introduced into a white‐walled room and their landing responses on large paired Coroplast squares were recorded. Parallel experiments 36 and 37 offered flies choices between squares that were not baited (blank) or baited with 31 glue‐mounted female stable flies (Exp. 25), or wingless 3‐dimensional (3D) fly decoys (Exp. 37). Small grey symbols show the data of individual replicates and large black symbols the mean (± SEM). Mean absolute numbers (± SEM) of landing responses are listed at the bottom of each jitter plot. Asterisks (*) denote a significant preference for stimulus 2 (***p <0.001, **p < 0.01; likelihood ratio tests).

Similar results were obtained in parallel experiments 36–37 that were run in a large room and offered bioassay flies a choice between an unbaited square and a square that was baited with either 10 flies (Exp. 36) or with 10 3D fly decoys (Exp. 37). In both experiments, significantly more flies landed on baited squares [Exp. 36 (*n* = 10): 231 ± 30 vs. 59 ± 10; *F* = 77.477, *p* < 0.0001; Exp. 37 (*n* = 10): 119 ± 22 vs. 68 ± 6; *F* = 13.516, *p* = 0.0051; Figure 11].

In combination, these data indicate that 3D fly decoys can substitute stable flies for the attraction of foraging stable flies.

## DISCUSSION

Our data support the hypothesis that the presence of stable flies on a potential landing site context‐dependently affects landing decisions by foraging conspecific flies (Exps. 1–2; Figure 2). Female and male flies landed on such a site only when they were food‐deprived and CO_2_‐activated (Exps. 3–5; Figure 2). Decisions of foraging flies to land on a site were prompted by the presence of con‐ or heterospecific flies (Exps. 21–23; Figure 7), or fly‐like objects (Exps. 34, 37; Figures 10 and 11), provided they resembled stable flies. Decisions to land on a site were not affected by the sex or abundance of flies used as baits (Exps. 1–2, 13–20; Figures 2, 5, 6), their relative orientation on a site (Exps. 26–29; Figures 8 and S2) or the integrity of their wings (Exps. 27, 30; Figure 8, S2 and S4). Whether bait flies resided on a black or white surface (Exps. 7–9; Figure 3), with vertical or horizontal orientation (Exps. 10–12; Figures 4 and S1), did not affect landing decisions by foraging flies. Overall, our findings support the hypothesis that foraging stable flies visually orient towards conspecific‐like objects, a behaviour potentially linked to conspecific cueing.

Our findings that foraging stable flies were attracted to ‘bait flies' contrast with those in a previous report that stable flies avoided landing on adhesive traps with con‐ or heterospecific fly captures (Beresford & Sutcliffe, 2017). These contrasting results may be due to differences in the experimental design, such as the presentation of bait flies on non‐adhesive experimental squares in our study and the presence of fly captures on adhesive trap surfaces in the Beresford‐and‐Sutcliffe study. Alternatively, this disparity may be due to differences in the physiological state of responding flies. In the 2017 study, the traps were affixed to the wall of a calf barn, and the flies that avoided traps may have recently fed on the calves and were no longer foraging. Non‐foraging stable flies preferentially perch on prominent objects which facilitate mate encounters or aid in thermoregulation (Bishopp, 1913; Buschman & Patterson, 1981). Males on these prominent perches exhibit aggressive behaviours, driving off smaller males and harassing passing females (Buschman & Patterson, 1981). It follows that non‐foraging stable flies are less likely to aggregate and may instead select perching sites with few, if any, conspecifics present.

Conversely, as demonstrated in our study, actively foraging stable flies were attracted to conspecifics (Exps. 1–5; Figure 2). Stable flies generally reside on a host only when they are actively feeding (Rochon et al., 2021). Therefore, conspecifics on a host may signal a host's suitability. For example, *Amblyomma hebraeum* ticks successfully feeding on a host release pheromone that informs conspecifics about the host's suitability as a food resource (Norval et al., 1989). Moreover, foraging flies being attracted to, and feeding in concert with, conspecifics may increase feeding efficiency (Tripet et al., 2009). In sand flies, group‐feeding elevates the concentration of immunosuppressive salivary agents, lowers the saliva expenditure for each fly in the group, shortens feeding bouts and increases female fecundity (Tripet et al., 2009). Stable fly saliva likewise contains factors which promote vasodilation in the host, slow blood‐clotting and dose‐dependently inhibit the proliferation of bovine lymphocytes (Olafson et al., 2021; Swist et al., 2002). Further investigation is warranted to determine whether blood‐feeding stable flies, like sand flies, obtain fitness benefits from cooperative feeding.

Although group‐feeding may accrue benefits to blood‐feeding flies, it may also increase resource competition. Yet, we found no evidence that foraging flies selected sites based on the relative abundance of flies already present on sites. While foraging flies preferred sites with conspecifics present, they did not discern between sites with one or 10 conspecifics, 10 or five conspecifics, 10 or 20 conspecifics (Figures 5, 6 and S3), or with flies in clustered or uniform spacing (Figure S5). Generally, attraction to conspecifics is most strongly selected for when (*i*) individuals benefit from the association and (*ii*) resource patches are not easily exhaustible (Prokopy & Roitberg, 2001). The latter concept is likely true for stable flies. Once located, host animals are generally a persistent resource, with host fatalities due to excessive stable fly feeding occurring only during severe fly outbreaks (e.g., Bishopp, 1913). With increasing biting fly abundance, defensive behaviours of cattle intensify (Hansen et al., 2023; Odeniran & Ademola, 2018; Torr & Mangwiro, 2000) which, in turn, diminish the flies' feeding success and blood meal size (Waage & Nondo, 1982). However, sustained large stable fly abundance may also reduce the expression of host‐defensive behaviours as cattle habituate to stable fly attacks over time (Dougherty et al., 1993; Mullens et al., 2006). Given that pain habituation is a function of stimulus intensity, frequency and localization (Jepma et al., 2014; Milne et al., 1991), cattle parasitized by flies may habituate more rapidly if flies aggregate on specific on‐host feeding sites. As host selection by stable flies and mosquitoes is affected by the frequency of host‐defensive behaviours (Edman & Scott, 1987; Warnes & Finlayson, 1987), a large cohort of flies on a host may guide foraging flies to a particularly placid and thus suitable host.

If stable flies—as mechanical vectors of disease‐causing pathogens and parasites (Baldacchino et al., 2013)—were to form feeding aggregations on particularly attractive host animals, this may affect the transmission dynamics of these disease‐causing agents. If we were to accept the model prediction that highly infectious and/or susceptible host animals sustain pathogen transmission chains in a herd population (Doeschl‐Wilson et al., 2021), it follows that host animals preferentially fed on by stable flies may, analogously, sustain similar transmission chains.

To foraging stable flies, the attractiveness of bait flies was not linked to their species‐specific characteristics (e.g., Exp. 23), motion, relative orientation or intact wings (Exps. 6, 26–30; Figures 2, 6 and S2). As long as bait flies, such as house flies, resembled stable flies they attracted stable flies (Exps. 23–25; Figure 7). Conversely, bait flies that were much smaller (horn flies) or larger (black blow flies) than stable flies failed to attract stable flies (Exps. 24–25; Figure 5). In an oviposition context, gravid female stable flies avoid sites bearing the chemical and microbial cues of heterospecific flies (Baleba et al., 2020; Hennig et al., 2024). However, in a foraging context, as demonstrated here with CO_2_‐stimulated food‐deprived flies, heterospecific house flies as baits attracted foraging stable flies. As house flies are not haematophagous, the attraction of stable flies to house flies is not likely due to foraging associations between house flies and stable flies. Instead, stable flies may be attracted to house flies simply because they seem unable to recognize the subtle visual characteristics that separate stable flies from house flies. This interpretation is supported by data showing that even three‐dimensional fly‐look‐alike decoys elicited stable fly attraction (Exps. 33–37; Figures 10 and 11). Both foraging and mate‐seeking house flies, and territory‐defending or mate‐seeking male stable flies, approach dark fly‐sized decoys (Buschman & Patterson, 1981; Collins & Bell, 1996; Tobin & Stoffolano Jr., 1973), suggesting that these muscid flies share some conspecific recognition cues in multiple behavioural contexts.

As comparatively more stable flies were attracted to conspecifics than to fly decoys when they were presented in a large bioassay room instead of a small bioassay cage (Exps. 36–37; Figure 11), it seems that bait stable flies offer additional mid‐range cues that decoys lack. We predicted that these mid‐range cues pertained to either the spacing pattern of bait flies on the larger squares or to the presence of wings. However, bait flies clustered closely together did not elicit a differential response from bait flies uniformly distributed across a square (Exp. S9; Figure S5), and bait flies with ablated wings remained attractive to foraging flies (Exp. 30; Figure 8). The rather indiscriminate responses of bioassay flies to bait flies, their density and distribution pattern may be due to the physiological constraints of the flies' eyes. The compound eyes of flies are attuned to motion sensing, trading off visual acuity for high temporal resolution (Kirschfeld, 1976).

Odour cues of bait stable flies, on their own, failed to attract foraging stable flies (Figure 9a, Exp. 31). Thus, the mechanisms underlying the attraction of foraging stable flies to conspecific flies, as demonstrated in our study, differ from those underlying the ‘fly factor phenomenon’, where food currently or previously fed on by flies attracts more flies than the same food kept inaccessible to flies (Barnhart & Chadwick, 1953). Whereas this ‘fly factor phenomenon’ is exclusively odour‐based (Carlson et al., 2000; Holl & Gries, 2018), olfactory and visual cues in combination may attract more stable flies than either cue on its own. Odour cues may arise during blood‐feeding. For example, Western tree hole mosquitoes, *Aedes sierrensis*, preferentially oriented to guinea pig hosts that were actively fed on by (visually occluded) conspecifics (Ahmadi & McClelland, 1985). Whether the blood‐feeding mosquitoes themselves emitted the odour or they increased host kairomone emission as a byproduct of their feeding activity is not clear. Blood‐feeding sand flies release an aggregation pheromone that prompts cooperative feeding (Schlein et al., 1984). If blood‐feeding stable flies were also to emit a pheromone, synthetic pheromone presented together with both attractive fly visual cues (this study) and attractive host thermal and olfactory cues (Hung et al., 2024; Kovacs et al., 2024) could be exploited for stable fly control.

In conclusion, we describe context‐dependent attraction of stable flies to conspecific flies, a phenomenon not previously known. Landing responses of CO_2_‐activated, food‐deprived stable flies were triggered by the presence of con‐ or heterospecific flies, or fly‐like objects, provided they resembled stable flies. Trapping systems for biting flies commonly present host‐like visual or olfactory cues that attract flies (e.g., Zhu et al., 2016). Our findings demonstrate that fly‐like cues, alone or in combination with host‐like cues, may increase the effectiveness of stable fly trapping systems.

## AUTHOR CONTRIBUTIONS

**Emmanuel Hung:** Conceptualization; investigation; methodology; writing – original draft; writing – review and editing; visualization; formal analysis; data curation. **Justin Wong:** Investigation; writing – original draft. **Augustus Negraeff:** Investigation. **Anya Gould:** Investigation. **Gerhard Gries:** Supervision; conceptualization; writing – review and editing; funding acquisition.

## FUNDING INFORMATION

EH was supported by Graduate Fellowships from SFU, a Master of Pest Management Graduate Entrance Scholarship, a Thelma Finlayson Graduate Entrance Scholarship, a Mutual Fire Insurance Company of BC Graduate Scholarship, and an Alexander Graham Bell Scholarship from the Natural Sciences and Engineering Research Council of Canada (NSERC). The research was supported by an NSERC—Industrial Research Chair to GG, with BASF Canada Inc. and Scotts Canada Ltd. as the industrial sponsors.

## CONFLICT OF INTEREST STATEMENT

The authors declare that the research was conducted in the absence of any commercial or financial relationships that could be construed as a potential conflict of interest.

## Supporting information


**Data S1.** Supporting Information.

## Data Availability

The datasets analysed for this study, and their associated code are publicly available and can be found in Mendeley Data at https://doi.org/10.17632/jm67zdr78k.1.
