# Supplementary material for: Conspecific cueing or cooperative feeding?—Foraging stable flies are visually attracted to conspecific flies
Source: Med Vet Entomol. 2026 Mar 13;40(3):512–29. doi: 10.1111/mve.70057 (PMC13436533; doi:10.1111/mve.70057)
Supplement: Supplementary file 1 [file MVE-40-512-s001.pdf]

Table S1. List of supplementary laboratory behavioral experiments (Exp.), the number of replicates (*n*) run, and the bioassay settings in which paired stimuli were presented to female bioassay flies[1].

**Exp. # (*n*)**	**Bioassay setting[2]**	**Stimulus 1**	**Stimulus 2**
S1 (10)	cage + CO_2[3]	horizontal (horiz.) black square with 10 dead stable fly females	horiz. black square with 20 dead stable fly females
S2 (10)	cage + CO_2[3]	horiz. black square with 10 dead stable fly females	horiz. black square with 20 dead stable fly females
S3 (10)	cage + CO_2[3]	horiz. black square with 10 dead stable fly females (oriented uniformly)	horiz. black square with 10 dead stable fly females (oriented randomly)
S4 (10)	cage + CO_2[3]	horiz. black square with 10 live stable fly females (mounted ventral side up)	horiz. black square with 10 live stable fly females (mounted dorsal side up)
S5 (10)	cage + CO_2[3]	horiz. black square with 10 dead stable fly females (mounted ventral side up)	horiz. black square with 10 dead stable fly females (mounted dorsal side up)
S6 (10)	cage + CO_2[3]	horiz. black square with 10 live stable fly females (mid-legs ablated)	horiz. black square with 10 live stable fly females (wings ablated)
S7 (10)	cage + CO_2[3]	horiz. black square with 10 dead stable fly females (mid-legs ablated)	horiz. black square with 10 dead stable fly females (wings ablated)
S8 (10)	3-chamber olfactometer	petri dish visually occluded by four layers of cheese cloth	petri dish with moist cotton ball visually occluded by four layers of cheese cloth
S9 (10)	room + CO_2[3]	vertical (vert.) black square with 31 dead stable fly females (uniformly spaced over the entire square)	vert. black square with 31 dead stable fly females (clustered in square center)
S10 (10)	room + CO_2[3]	vert. black square with 31 dead stable fly females (mid-legs ablated)	vert. black square with 31 dead stable fly females (wings ablated)

[1] All lab-reared bioassay stable flies were 3- to 7-day-old (i.e., days post eclosion) at the time of testing and were starved 20–24 h prior to experimentation.

[2] Two-choice bioassays were run in a metal mesh cage (‘cage’; 45 × 45 × 45 cm; Figure 1B), a 3-chamber still-air olfactometer (‘3-chamber olfactometer’; 45 × 21 × 15 cm; Figure 9B), or a white-walled room (‘room’; 2.9 m × 2.5 m × 2 m).

[3] Flies were tested at elevated levels of CO_2 released from dry ice (30 g) in an open petri dish in the center of the cage ceiling (Figure 1B).

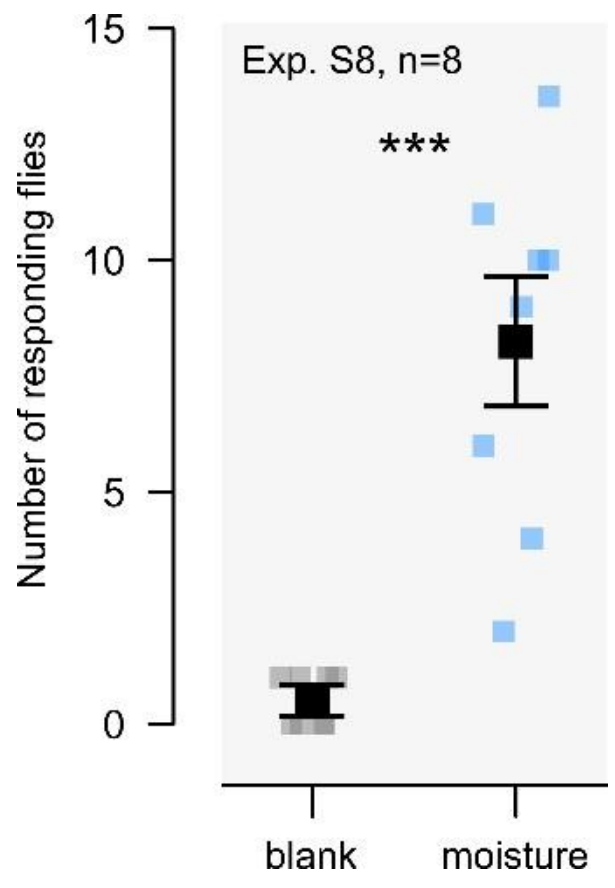

Figure S1. Attraction of foraging stable flies to a moisture cue. In each 6-h experimental replicate (*n*), 15 food- and water-deprived female flies were introduced into the central chamber of a 3-chamber olfactometer (Figure 9B) from which they could enter, but not exit, lateral chambers housing test stimuli. Small gray and blue symbols show the data of individual replicates and large black symbols the mean (± SEM) number of responding flies. Asterisks (*) denote a significant preference (*** $p < 0.001$). More foraging flies entered the lateral chamber baited with a moist cotton ball than the unbaited lateral chamber [Exp. S8 ($n = 8$): 8.25 ± 1.40 vs. 0.5 ± 0.189; $t(7) = -6.2$, $p = 0.0004$; two-sample t-test], suggesting that moisture (relative humidity) is a host-foraging or host-recognition cue.

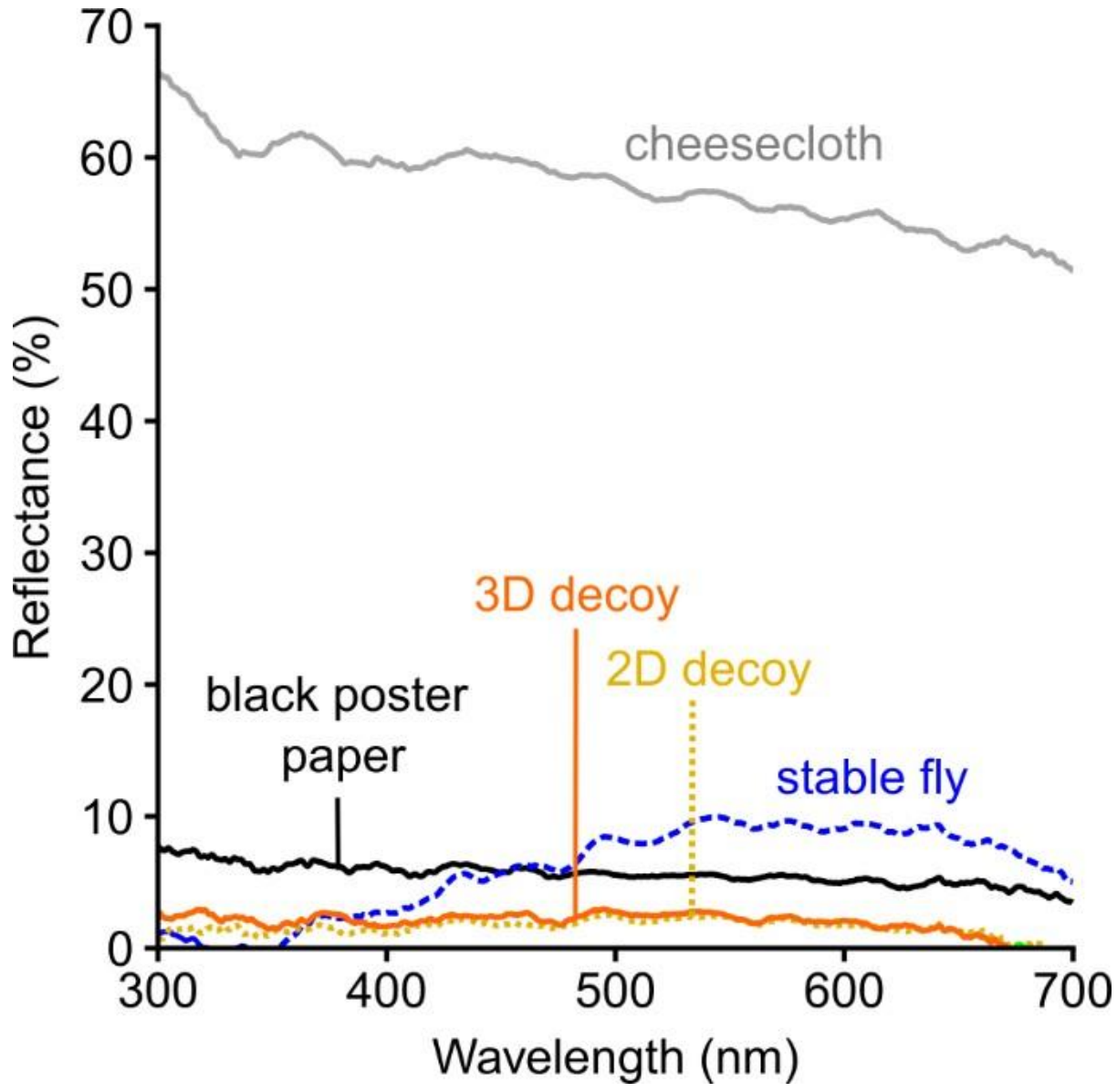

Figure S2. Reflectance spectra of test stimuli used in behavioral experiments. Female stable flies (dashed blue line), three-dimensional (3D) black plastic decoys (solid orange line), and two-dimensional (2D) black plastic decoys (dotted yellow line) were glue-mounted onto squares of black poster paper (solid black line). To visually occlude bait flies, stimulus squares were covered with either perforated black poster paper (Exp. 31) or four layers of white cheese cloth (Exp. 32; solid grey line).

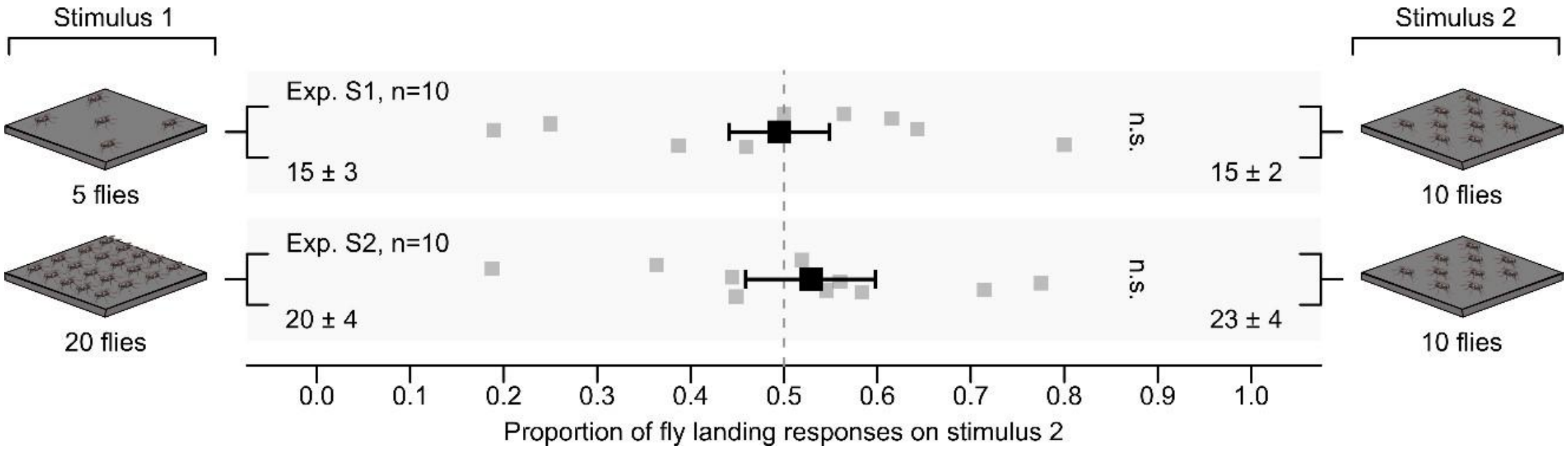

Figure S3. Effects of female stable fly abundance on squares on foraging decisions of female stable flies. In each 5-min experimental replicate (*n*), 25 food-deprived, CO_2-stimulated flies were introduced into a bioassay cage and their landing responses on paired horizontally-oriented Coroplast squares (Fig. 1A,B) were recorded. Bioassay flies were offered a choice between squares that were baited with 10 or 5 glue-mounted female flies (Exp. S1), and 10 or 20 glue-mounted female flies (Exp. S2). Small gray symbols show the data of individual replicates and large black symbols the mean (± SEM). Mean absolute numbers (± SEM) of alighting responses are listed at the bottom of each jitter plot; 'n. s.' denotes no significant preference ($p > 0.05$; likelihood ratio tests).

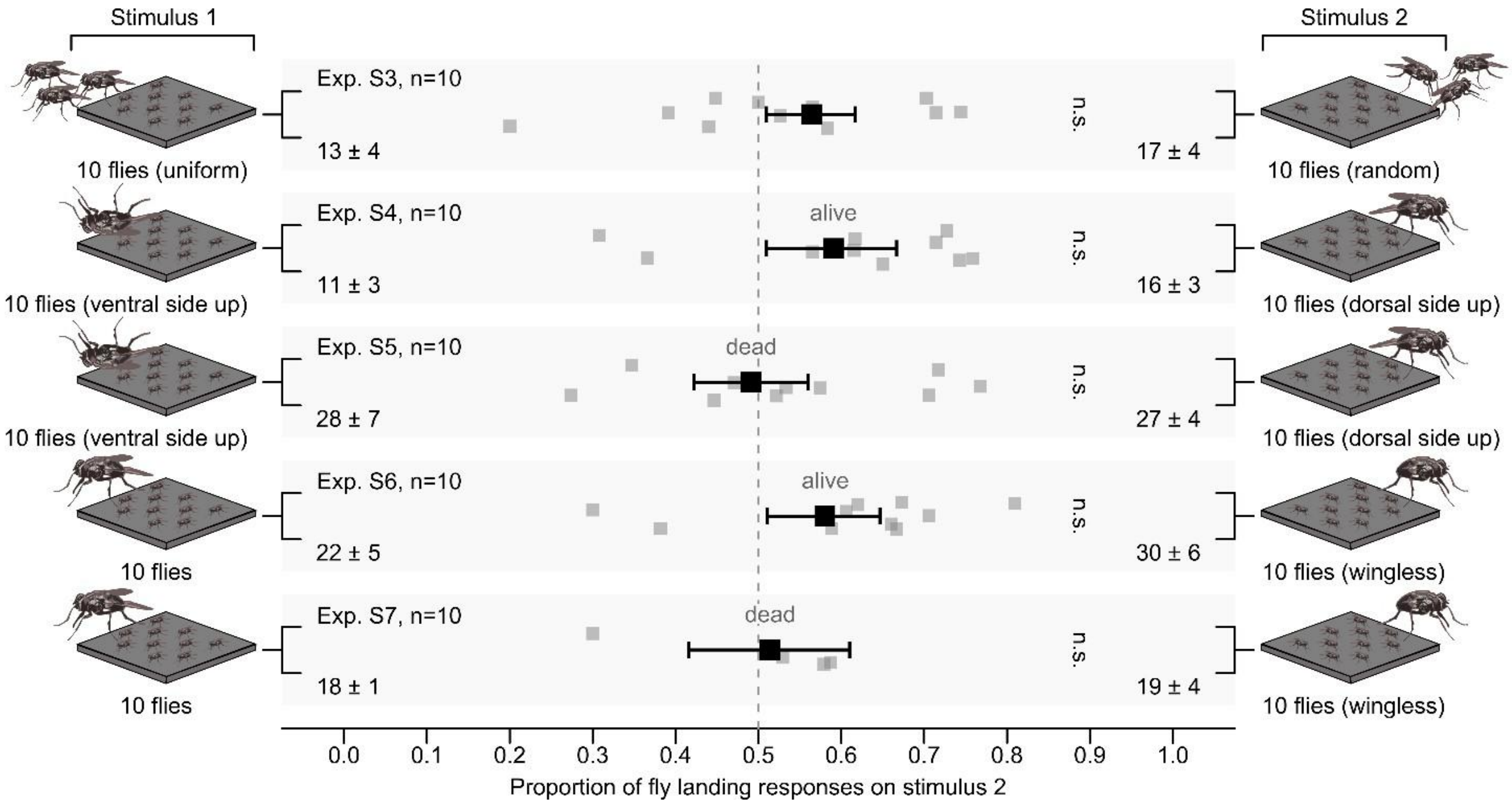

Figure S4. Visual effects of female stable flies glue-mounted as bait flies on squares on foraging decisions of female stable flies. In each 5-min experimental replicate (*n*), 25 food-deprived, CO_2-stimulated female flies were introduced into a bioassay cage and their landing responses on paired horizontally-oriented Coroplast squares (Fig. 1A,B) were recorded. In experiment S3, flies were offered a choice between two squares each baited with 10 flies, all pointing in the same (uniform) direction or all pointing randomly in various directions. Experiments S4 and S5 offered a choice between bait flies that were mounted ventral-side-up or dorsal-side-up, using live females (Exp. S4) or dead females (cold-euthanized < 1 h prior to testing; Exp. S5). Experiments S6 and S7 offered a choice between bait flies with intact or ablated wings, using live females (Exp. S6) or dead females (Exp. S7). Small gray symbols show the data of individual replicates and large black symbols the mean (± SEM). Mean absolute numbers (± SEM) of landing responses are listed at the bottom of each jitter plot; 'n. s.' denotes no significant preference ($p > 0.05$; likelihood ratio tests).

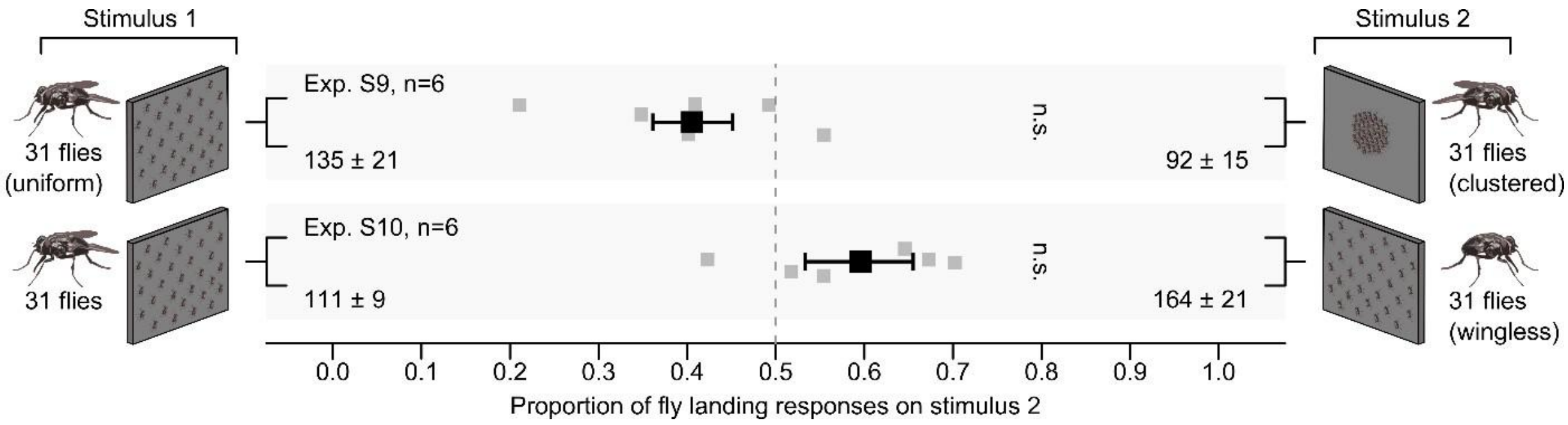

Figure S5. Effects of female stable flies mounted on squares on foraging decisions of female stable flies in whole-room bioassays. In each 5-min experimental replicate (*n*), 50 food-deprived, CO_2-stimulated female flies were introduced into a white-walled room and their landing responses on large paired Coroplast squares (each 25 cm × 25 cm) were recorded. Flies were offered a choice between squares that were baited with 31 glue-mounted female stable flies that were either uniformly spaced across the entire square ('uniform') or tightly clustered in the square center ('clustered') (Exp. S9), or that were uniformly spaced and with their wings intact or ablated (Exp. S10). Small gray symbols show the data of individual replicates and large black symbols the mean (± SEM). Mean absolute numbers (± SEM) of landing responses are listed at the bottom of each jitter plot. 'n. s.' denotes no significant preference ($p > 0.05$; likelihood ratio tests).
